# ﻿Phylogeny and taxonomy of *Nigroporus* (Polyporales, Basidiomycota) with four new species from Asia and Oceania

**DOI:** 10.3897/mycokeys.112.127011

**Published:** 2025-01-20

**Authors:** Xiang-Lin Li, Yu-Cheng Dai, Zhan-Bo Liu, Yu-Han Jiang, Hong-Gao Liu, Yuan Yuan

**Affiliations:** 1 State Key Laboratory of Efficient Production of Forest Resources, School of Ecology and Nature Conservation, Beijing Forestry University, Beijing, 100083, China Beijing Forestry University Beijing China; 2 Yunnan Key Laboratory of Gastrodia and Fungi Symbiotic Biology, Zhaotong University, Zhaotong, 657000, China Zhaotong University Zhaotong China

**Keywords:** Biodiversity, molecular systematics, Steccherinaceae, taxonomy, wood-inhabiting fungi

## Abstract

*Nigroporusvinosus* (Berk.) Murrill, first described from North America, was considered to be a common species in China. The existence of a species complex is confirmed through a phylogenetic analysis of samples examined. Based on morphological examination and molecular evidence, four new species are described as *Nigroporusaustralianus*, *N.austroasianus*, *N.subvinosus* and *N.yunnanensis*. They are characterized by pileate, effused-reflexed to resupinate, purplish, vinaceous to brown basidiomata when fresh, mostly becoming brown when dry. *Nigroporusaustralianus* is characterized by narrower basidiospores measuring 3.4–4.1 × 1.3–1.5 µm, thicker contextual hyphae measuring 3.2–6.4 µm in diam and a geographical distribution in Australia. *Nigroporusaustroasianus* is characterized by smaller pores measuring 10–13 per mm, generative hyphae dominant in the tube trama, small basidiospores measuring 3–4.1 × 1.5–2 μm and a distribution in Malaysia and tropical to subtropical regions of China. *Nigroporussubvinosus* is characterized by skeletal hyphae with thin to slightly thick walls, barrel- to pear-shaped basidia, and long cystidioles measuring 10–18 µm and is common in Asia. *Nigroporusyunnanensis* is characterized by thinner pilei measuring 2.5 mm thick at the base, bigger basidiospores measuring 4–4.5 × 1.9–2.2 μm and is found only in Yunnan. The 2-gene (ITS+nLSU) analysis of the Steccherinaceae indicated that the four new species nested in the *Nigroporus* clade. The 3-gene (ITS+nLSU+*TEF1*) analysis of the genus *Nigroporus* showed that *N.australianus* formed a monophyletic lineage, *N.subvinosus* was sister to *N.austroasianus* and *N.yunnanensis*, and *N.austroasianus* was sister to *N.yunnanensis.* Furthermore, *N.vinosussensu stricto* is also distributed in Asia.

## ﻿Introduction

The genus *Nigroporus* (Steccherinaceae, Polyporales), typified by *N.vinosus* (Berk.) Murrill, was established by Murrill (1905). It is characterized by stipitate, pileate, effused-reflexed to resupinate, pinkish or violet to dark bluish gray basidiomata when fresh, a dimitic hyphal system with fuliginous skeletal hyphae and clamped generative hyphae, allantoid to cylindrical basidiospores usually less than 5 µm long, growing mostly on angiosperms and occasionally on gymnosperms, causing a white rot and having a distribution mainly in the subtropical and tropical forests of the world (Ryvarden 2004; Dai 2009, 2012; Hattori et al. 2012; Ryvarden et al. 2022; Wu et al. 2022; Zhao et al. 2024). As wood-inhabiting fungi, species of *Nigroporus* are saprophytes (Yuan et al. 2023), but they play a special role in forest ecosystems by decomposing wood and recycling organic matter (Hattori and Lee 2003; Wei and Dai 2004; Olou et al. 2023). Phylogenetic analysis demonstrated that *Nigroporus* belonged to the family Steccherinaceae in the Polyporales (Justo et al. 2017).

Previously, four species were accepted in the genus: *Nigroporusmacroporus* Ryvarden & Iturr., *N.stipitatus* Douanla-Meli & Ryvarden, *N.ussuriensis* (Bondartsev & Ljub.) Y.C. Dai & Niemelä and *N.vinosus* (Dai and Niemelä 1995; Ryvarden and Iturriaga 2003; Douanla-Meli et al. 2007; Ryvarden et al. 2022). The molecular data of *N.stipitatus* and *N.vinosus* are available (Miettinen et al. 2012; Binder et al. 2013; Piepenbring et al. 2020). In addition, Ko and Jung (1999) mentioned that *Trametesconsors* (Berk.) A. Mitra is more closely related to *Nigroporus* than *Trametes*.

Due to the lack of molecular data for *N.macroporus* and *N.ussuriensis*, the phylogenetic relationship among taxa of the genus was poorly known. During recent investigations on polypores in Asia and Oceania, more samples were collected and their morphology corresponds to the concept of *N.vinosus*. To confirm their identification, morphological examination and phylogenetic analyses based on the internal transcribed spacer (ITS), large subunit nuclear ribosomal RNA (nLSU) and translation elongation factor 1-alpha (*TEF1*) genes were carried out. Four new species are confirmed in our studied samples. All these four species are different from *N.vinosussensu stricto*, and they are described as new species in this paper.

## ﻿Materials and methods

### ﻿Morphological studies

The studied specimens are deposited at the Fungarium of Beijing Forestry University (BJFC). Microscopic procedure followed Wu et al. (2022), and color terms followed Anonymous (1969) and Petersen (1996). Sections were studied at a magnification of up to 1000× using a Nikon E 80i microscope with phase-contrast illumination. Measurements and description of microscopic structures were made from slide preparations stained with Cotton Blue (CB) and Melzer’s reagent (IKI). Basidiospores were measured from sections cut from the tubes. To represent the variation in the size of the basidiospores, 5% of the measurements were excluded from each end of the range and are given in parentheses. Abbreviations used are as follows: KOH = 5% potassium hydroxide solution, IKI– = neither amyloid nor dextrinoid, CB– = acyanophilous, L = mean basidiospore length (arithmetic average of all basidiospores), W = mean spore width (arithmetic average of all basidiospores), Q = variation in the ratios of L/W between specimens studied, n = number of basidiospores measured from a given number of specimens.

### ﻿DNA extraction, amplification, and sequencing

A Cetyltrimethylammonium Bromide (CTAB) new type plant DNA kit (Aidlab Biotechnologies Co., Ltd, Beijing, China) was used to obtain DNA from dried specimens, followed by the polymerase chain reaction (PCR) according to the manufacturer’s instructions with some modifications as described by Mao et al. (2023). Amplification of the ITS region used primer pairs ITS5 and ITS4 (White et al. 1990). Amplification of the nLSU regions used primer pairs LR0R and LR7 (Vilgalys and Hester 1990). Amplification of part of *TEF1* used primer pairs EF1-983F and EF1-1567R (Rehner and Buckley 2005). The PCR cycling schedule for ITS was as follows: initial denaturation at 95 °C for 3 min, followed by 35 cycles at 94 °C for 40 s, 54 °C for 45 s, 72 °C for 1 min, and a final extension of 72 °C for 10 min. The PCR procedure for the nLSU was as follows: initial denaturation at 94 °C for 1 min, followed by 35 cycles at 94 °C for 30 s, 50 °C for 1 min, and 72 °C for 1.5 min, and a final extension of 72 °C for 10 min. The reaction procedure for the *TEF1* is the same as the ITS, except that the annealing temperature is adjusted to 58 °C respectively (Mao et al. 2023). The purification and sequencing of the PCR products was conducted by the Beijing Genomics Institute (BGI), Beijing, China, with the same primers as used in PCR. The PCR amplified DNA products were stored in a refrigerator at minus 20 °C and newly generated sequences were deposited at GenBank (Tables 1, 2). Sequences generated from this study were aligned with additional sequences downloaded from GenBank using BioEdit (Hall 1999) and ClustalX (Thompson et al. 1997). In this study, a 2-gene dataset (ITS+nLSU) and 3-gene dataset (ITS+nLSU+*TEF1*) were used to reconstruct the phylogenetic position of the new species. All sequences of ITS, nLSU, *TEF1* were respectively aligned in MAFFT 7 (https://mafft.cbrc.jp/alignment/server/; Katoh et al. 2019) and BioEdit. Alignments were spliced and exported to nexus and phylip in Mesquite (Maddison and Maddison 2021). The missing sequences and ambiguous nucleotides were coded as N.

**Table 1.** Information on the sequences used in this study. New sequences are in bold. * type specimen.

Species	Sample number	Location	GenBank accession No	
			ITS	nLSU
*Antellaamericana*	KHL 11949	Sweden	JN710509	JN710509
*A.americana*	HHB-4100	USA	KP135316	KP135196
*A.chinensis*	Dai 8874	China	JX110843	KC485541
*A.chinensis*	Dai 9019	China	JX110844	KC485542
*A.niemelaei*	Renvall 3218	Finland	AF126876	-
*A.niemelaei*	Haikonen 14727	Finland	AF126877	-
*Antrodiellaonychoides*	Miettinen 2312	Finland	JN710517	JN710517
*A.pallescens*	Nordén 8.8.2008	Sweden	JN710518	JN710518
*A.romellii*	Miettinen 7429	Finland	JN710520	JN710520
*A.semisupina*	Labrecque & Labbé 372	Canada	JN710521	JN710521
*A.stipitata*	FD-136	USA	KP135314	KP135197
*A.stipitata*	Yuan 5640	China	KC485525	KC485544
*Atraporiellaneotropica*	Miettinen X1021	Belize	HQ659221	HQ659221
*A.yunnanensis*	CLZhao 604	China	MF962482	MF962485
*A.yunnanensis*	CLZhao 605	China	MF962483	MF962486
*Butyreajaponica*	MN 1065	Japan	JN710556	JN710556
*B.luteoalba*	FP-105786	USA	KP135320	KP135226
*B.luteoalba*	KHL 13238b	Estonia	JN710558	JN710558
*Climacocystisborealis*	KHL 13318	Estonia	JN710527	JN710527
*Elaphroporiaailaoshanensis*	CLZhao 596	China	MG231572	MG748855
*E.ailaoshanensis*	CLZhao 597	China	MG231847	MG748856
*Etheirodonfimbriatum*	KHL 11905	Sweden	JN710530	JN710530
*E.fimbriatum*	HR 98811	Czech	MT849300	-
*E.purpureum*	MCW 642/18	Brazil	MT849301	MT849301
*Flaviporusbrownii*	MCW 362/12	Brazil	KY175008	KY175008
*F.brownii*	X 462	Australia	JN710538	JN710538
*F.liebmannii*	X 249	China	JN710539	JN710539
*F.liebmannii*	Yuan 1766	China	KC502914	-
*F.subundatus*	MCW 367/12	Brazil	KY175004	KY175004
*F.subundatus*	MCW 457/13	Brazil	KY175005	KY175005
*F.tenuis*	MCW 442/13	Brazil	KY175001	KY175001
*F.tenuis*	MCW 356/12	Brazil	KY175002	KY175002
*Frantisekiafissiliformis*	CBS 435.72	USA	MH860521	MH872232
*F.mentschulensis*	BRNM 710170	Czech	FJ496670	FJ496728
*F.mentschulensis*	AH 1377	Austria	JN710544	JN710544
*F.ussurii*	Wei 3081	China	KC485527	KC485545
*F.ussurii*	Dai 8249	China	KC485526	-
*Junghuhniacrustacea*	X 262	Indonesia	JN710553	JN710553
*J.delicate*	MCW 564/17	Brazil	MT849295	MT849295
*J.delicate*	MCW 693/19	Brazil	MT849297	MT849297
*J.pseudocrustacea*	Yuan 6160	China	MF139551	-
*J.pseudocrustacea*	Zhou 283	China	MF139552	-
*Loweomycesfractipes*	X 1149	Slovakia	JN710570	JN710570
*L.fractipes*	MT 13/2012	Brazil	KX378866	KX378866
*L.spissus*	MCW 488/14	Brazil	KX378869	KX378869
*L.tomentosus*	MCW 366/12	Brazil	KX378870	KX378870
*L.wynneae*	X 1215	Denmark	JN710604	JN710604
*Metuloideacinnamomea*	X 1228	Vietnam	KU926963	-
*M.fragrans*	LE 295277	Russia	KC858281	-
*M.murashkinskyi*	X 449	Russia	JN710588	JN710588
*M.reniformis*	MCW 542/17	Brazil	MT849303	MT849303
*M.reniformis*	MCW 523/17	Brazil	MT849302	MT849302
*M.rhinocephala*	X 460	Australia	JN710562	JN710562
*Mycorrhaphiumhispidum*	MCW 363/12	Brazil	MH475306	MH475306
*M.hispidum*	MCW 429/13	Brazil	MH475307	MH475307
*M.subadustum*	Yuan 12976	China	MW491378	MW488040
*M.subadustum*	Dai 10173	China	KC485537	KC485554
***Nigroporusaustralianus***	Cui 16775*	**Australia**	**PP622349**	-
***N.austroasianus***	**Dai 18594**	**Malaysia**	**PP622343**	**PP625976**
***N.austroasianus***	Dai 20632*	**China**	**PP622344**	**PP669796**
***N.austroasianus***	**Dai 28512**	**China**	**PQ327581**	**PQ327583**
*N.vinosus*	BHS2008-100	USA	JX109857	JX109857
*N.vinosus*	8182	USA	JN710575	JN710575
***N.vinosus***	**Cui 7701**	**China**	**PP622353**	-
***N.vinosus***	**Cui 7854**	**China**	**PP622352**	-
***N.vinosus***	**Dai 16966**	**China**	**PP692550**	**PP625980**
***N.vinosus***	**Dai 26323**	**China**	**PP622348**	**PP625978**
***N.vinosus***	**Dai 26461**	**China**	**PP622347**	**PP625979**
***N.vinosus***	**Dai 28194**	**China**	**PP955188**	**PP939654**
*N.vinosus*	KA17-0261	Korea	MN294801	-
*N.stipitatus*	X 546	Cameroon	JN710574	JN710574
*N.stipitatus*	KaiR 116	Benin	MT110231	-
***N.subvinosus***	**Cui 17526**	**China**	**PP622350**	
***N.subvinosus***	**Cui 18104**	**China**	**PP622339**	**PP669797**
***N.subvinosus***	**Dai 13136**	**China**	**PP622342**	-
***N.subvinosus***	**Cui 18097**	**China**	**PP622345**	-
***N.subvinosus***	**Dai 20445**	**China**	**PP622340**	**PP625977**
***N.subvinosus***	**Dai 25154**	**China**	**PP622351**	
***N.subvinosus***	Dai 26787*	**China**	**PP622346**	**PP669794**
***N.subvinosus***	**Dai 27441**	**China**	**PP939653**	**PP955187**
*N.subvinosus*	LE-BIN 5057	Vietnam	OR683766	-
***N.yunnanensis***	**Cui 18205**	**China**	**PP622341**	**PP669795**
***N.yunnanensis***	Dai 19116*	**China**	**PP622337**	**PP625974**
***N.yunnanensis***	**Dai 19870**	**China**	**PP622338**	**PP625975**
*N.yunnanensis*	CLZhao 4067	China	OR167780	-
*N.yunnanensis*	CLZhao 4767	China	OR167781	-
*Steccherinumbourdotii*	HR99893	Czech	MT849311	-
*S.bourdotii*	Saarenoksa 10195	Finland	JN710584	JN710584
*S.hirsutum*	CLZhao 4222	China	MW290040	MW290054
*S.hirsutum*	CLZhao 4523	China	MW290041	MW290055
*S.ochraceum*	KHL11902	Sweden	JN710590	JN710590
*S.ochraceum*	2060	Sweden	JN710589	JN710589
*S.subtropicum*	CLZhao 16901	China	OP799391	-
*S.subtropicum*	CLZhao 11059	China	OP799390	OP799377
*Trullellaconifericola*	Cui 2851	China	MT269764	-
*T.conifericola*	Yuan 12655	Vietnam	MT269760	MT259326
*T.conifericola*	Yuan 12657	Vietnam	MT269761	MT259327
*T.dentipora*	X 200	Venezuela	JN710512	JN710512
*T.duracina*	MCW 410/13	Brazil	MH475309	MH475309
*T.duracina*	RP 96	Brazil	MH475310	MH475310
*T.duracina*	Dai 20474	China	OL437266	OL434415
*Xanthoporussyringae*	Jeppson 2264	Sweden	JN710607	JN710607
*X.syringae*	AFTOL-ID 774	China	AY789078	AY684166

**Table 2.** Information on the sequences of *TEF1* used in this study. New sequences are in bold. * type specimen.

Species	Specimen number	GenBank accession numbers
		*TEF1*
***Nigroporusaustralianus***	Cui 16775*	**PP719863**
***N.austroasianus***	**Dai 18594**	**PP719857**
***N.austroasianus***	Dai 20632*	**PP719858**
***N.austroasianus***	**Dai 28512**	**PQ540992**
***N.subvinosus***	**Cui 18104**	**PP706386**
***N.subvinosus***	**Dai 13136**	**PP719856**
***N.subvinosus***	Dai 26787*	**PP719859**
*N.vinosus*	8182	JN710728
***N.vinosus***	**Cui 7584**	**PP719855**
***N.vinosus***	**Dai 16966**	**PP719862**
***N.vinosus***	**Dai 26323**	**PP719861**
***N.vinosus***	**Dai 26461**	**PP719860**
***N.vinosus***	**Dai 28194**	**PP975431**
***N.yunnanensis***	**Cui 18205**	**PP719855**

### ﻿Phylogenetic analyses

In the study, a sequence of *Climacocystisborealis* (Fr.) Kotl. & Pouzar obtained from GenBank was used as an outgroup to root trees in the ITS+nLSU analysis of Steccherinaceae. Sequences of *Trullelladuracina* (Pat.) Zmitr. and *Trullellaconifericola* T. Cao & H.S. were used as the outgroups to root trees in the ITS+nLSU+*TEF1* analysis (Miettinen et al. 2012; Dong et al. 2023). Alignment datasets were deposited in TreeBase (submission ID 31362, 31363; http://www.treebase.org). Maximum Likelihood (ML) and Bayesian Inference (BI) analyses were conducted based on these datasets following previous studies (Liu and Dai 2021; Dong et al. 2023). The best-fit evolutionary model for each gene fragment (ITS, nLSU, and *TEF1*) was selected individually by Hierarchical Likelihood Ratio Tests (HLRT) in MrModeltest 2.2 (Nylander 2004) after scoring 24 models of evolution for each fragment in PAUP* version 4.0b10 (Swofford 2002).

Sequences were analyzed using Maximum Likelihood (ML) with raxmIHPC-PTHREADS-SSE3 through raxmlGUI 2.0.0. (Edler et al. 2021). Branch support (BS) for Maximum Likelihood (ML) is the supporting values of each node obtained by running 1000 bootstrap replicates under the GTRGAMMA model. Bayesian Posterior Probabilities (BPP) were computed with MrBayes 3.1.2 (Ronquist and Huelsenbeck 2003; Nylander 2004). Four Markov chains were run for 2 M generations (2-gene dataset), and for 1.2 M generations (3-gene dataset) until the split deviation frequency value was less than 0.01, and trees were sampled every 100 generations. The first 25% of the sampled trees were discarded as burn-in and the remaining ones were used to reconstruct a majority rule consensus and calculate Bayesian Posterior Probabilities (BPP) of the clades. For each dataset, partitions were manually defined for each gene region based on trimmed segment lengths, and the best model was selected for each gene segment. All trees were viewed in FIGTREE v. 1.4.3 (http://tree.bio.ed.ac.uk/software/figtree/). Branches that received bootstrap support (ML ≥ 50%, BPP ≥ 0.90) were considered as significantly supported. The ML bootstrap supports ≥ 50% and BPP ≥ 0.75 are presented on topologies from ML analyses, respectively.

## ﻿Results

### ﻿Phylogenetic analyses

**Figure 1.**
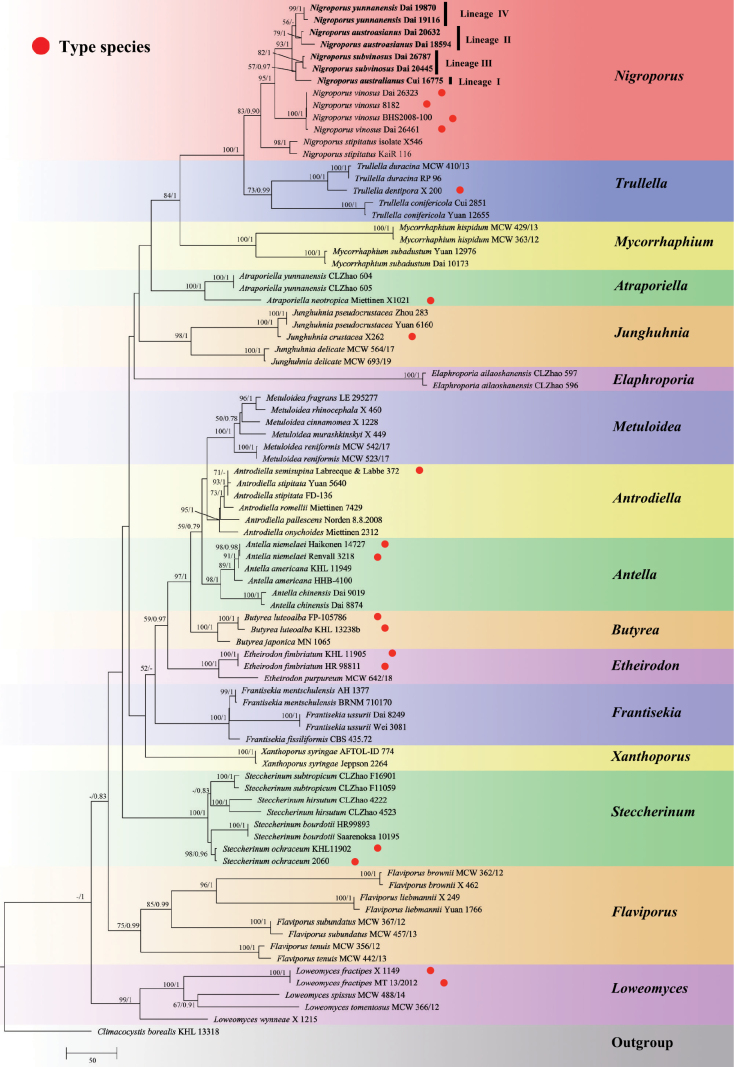
Maximum Likelihood strict consensus tree illustrating the phylogeny of four new species and related species in the family Steccherinaceae based on ITS+nLSU sequences. Branches are labeled with Maximum Likelihood bootstrap values higher than 50% and Bayesian Posterior Probabilities more than 0.75.

The combined 2-gene dataset (ITS+nLSU) included sequences from 85 samples representing 51 species. The dataset had an aligned length of 2265 characters, of which 1468 (65%) characters were constant, 173 (8%) were variable and parsimony-uninformative and 624 (27%) were parsimony informative. The phylogenetic reconstruction performed with ML and BI analyses for two combined datasets showed similar topology and few differences in statistical support. The best model-fit applied in the Bayesian analysis was GTR+I+G for both ITS+nLSU, lset nst = 6, rates = invgamma, and prset statefreqpr = dirichlet (1, 1, 1, 1). Bayesian analysis resulted in a nearly congruent topology with an average standard deviation of split frequencies = 0.009757 to ML analysis, and thus only the ML tree is provided (Fig. 1).

The phylogeny inferred from the ITS+nLSU sequences demonstrated that sixteen genera nested in the family Steccherinaceae and newly examined seven samples formed four distinct clades at the species level, nested in the *Nigroporus* clade (Marked as Lineage I–IV, Lineage I with 57% ML-BS, 0.97 BPP; Lineage II with 79% ML-BS, 1.00 BPP; Lineage III with 82% ML-BS, 1.00 BPP; Clade IV with 99% ML-BS, 1.00 BPP; Fig. 1).

**Figure 2.**
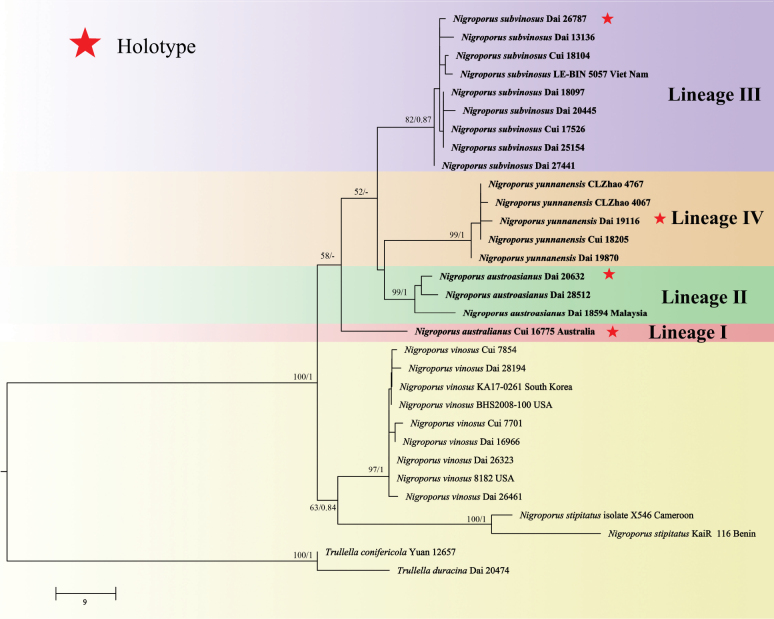
Maximum Likelihood analysis of *Nigroporus* based on a dataset of ITS+nLSU +*TEF1*. ML bootstrap values higher than 50% and Bayesian Posterior Probabilities values more than 0.75 are shown. New taxa are in bold.

The combined 3-gene dataset (ITS+nLSU+*TEF1*) included sequences from 31 samples representing eight species. The dataset had an aligned length of 2572 characters, of which 2317 (90%) characters were constant, 80 (3%) were variable and parsimony-uninformative and 175 (7%) were parsimony informative. The phylogenetic reconstruction performed with ML and BI analyses for two combined datasets showed similar topology and few differences in statistical support. The best model-fit applied in the Bayesian analysis were HKY+G for ITS (lset nst = 2, rates = gamma, and prset statefreqpr = dirichlet (1, 1, 1, 1)), GTR+G for nLSU (lset nst = 6, rates = gamma, and prset statefreqpr = dirichlet (1, 1, 1, 1)), SYM for *TEF1* (lset nst = 6 and prset statefreqpr = dirichlet (1, 1, 1, 1)). Bayesian analysis resulted in a nearly congruent topology with an average standard deviation of split frequencies = 0.009991 (BI), and thus only the ML tree is provided (Fig. 2). The phylogeny inferred from ITS+nLSU+*TEF1* sequences demonstrated that Lineage I formed a monophyletic lineage, Lineage II was sister to Lineage IV, and Lineage III was sister to Lineage II and Lineage IV (Fig. 2).

### ﻿Taxonomy

#### ﻿ Nigroporus australianus

Taxon classificationFungiPolyporalesSteccherinaceae

Y.C. Dai, X.L. Li & Yuan Yuan sp. nov.

3512BD93-AC62-5FB0-BA0D-3D93C96D588D

853914

Figs 3 , 4

##### Holotype.

Australia • Queensland, Cairns, Crater Lakes National Park, on fallen angiosperm trunk, 17 May 2018, *Cui 16775* (BJFC 030074).

##### Etymology.

*Australianus* (Lat.): refers to the species being found in Australia.

##### Description.

***Basidiomata*.** Annual, pileate, solitary to imbricate, leathery and without odor or taste when fresh, becoming woody hard and light in weight upon drying; pilei semicircular to flabelliform, projecting up to 3.4 cm, 7.2 cm wide and 3.5 mm thick at base. Pileal surface bay to purplish chestnut when fresh, becoming grayish brown to fuscous upon drying, concentrically zonate, glabrous, margin thin. Pore surface vinaceous when drying; sterile margin indistinct; pores round to angular, 9–10 per mm; dissepiments thin, lacerate. Context fawn, corky when dry, up to 0.5 mm thick. Tubes chestnut, corky when dry, up to 3 mm long.

**Figure 3.**
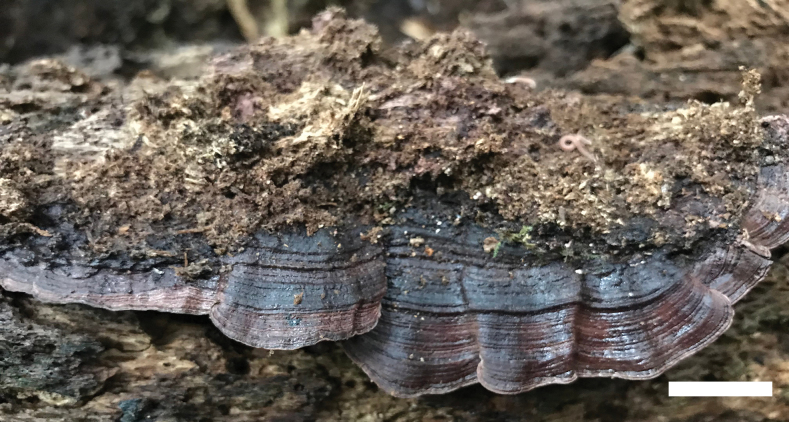
A basidioma of *Nigroporusaustralianus* (holotype, Cui 16775). Scale bar: 1 cm.

***Hyphal structure*.** Hyphal system dimitic; generative hyphae bearing clamp connections; skeletal hyphae IKI–, CB–; tissues darkening in KOH.

***Context*.** Generative hyphae hyaline, thin- to slightly thick-walled, occasionally branched, 3.2–6.4 µm in diam.; skeletal hyphae yellowish brown, thick-walled with a wide to narrow lumen, unbranched, slightly flexuous, interwoven, 3.2–5.4 µm in diam.

**Figure 4.**
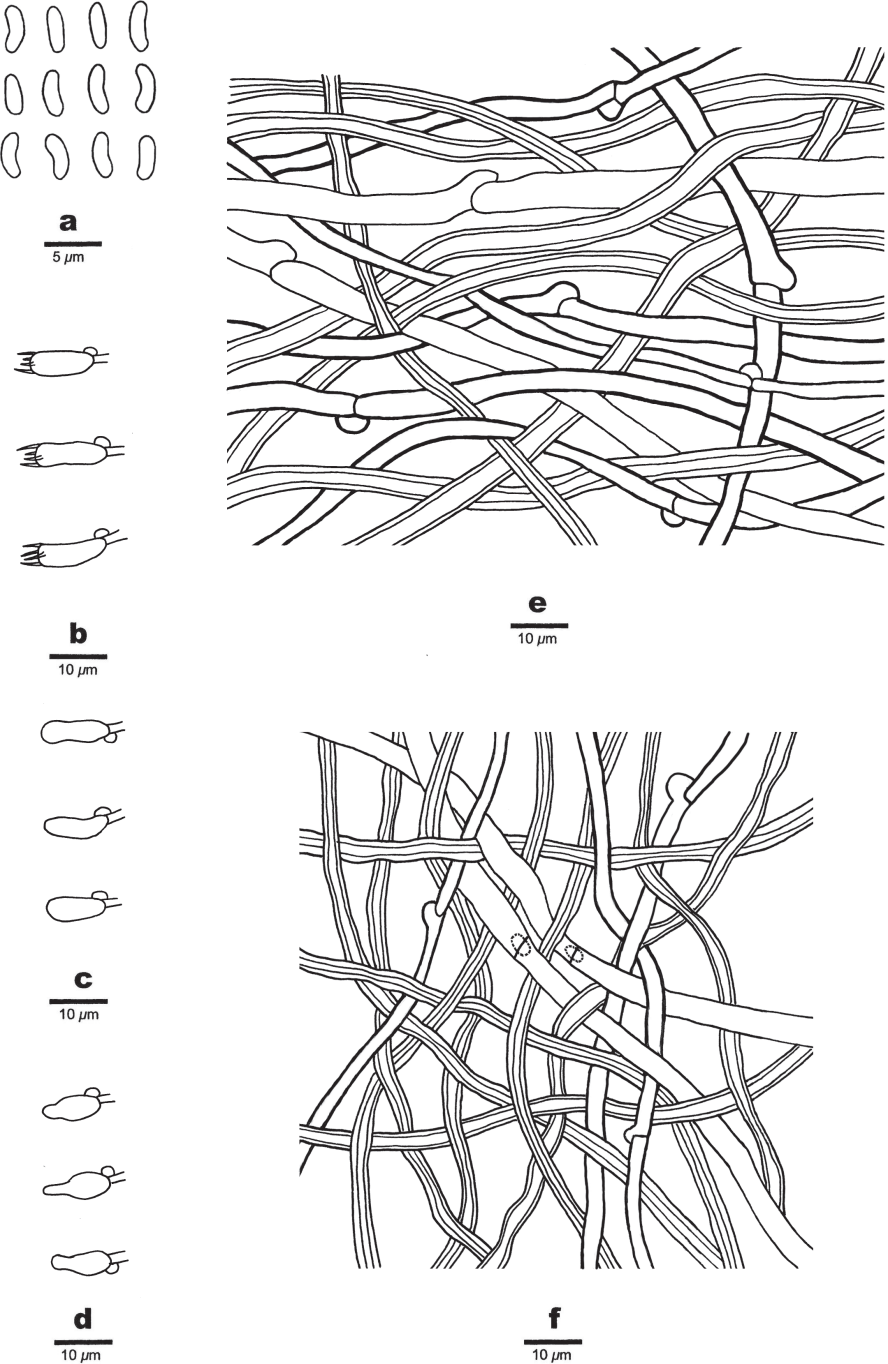
Microscopic structures of *Nigroporusaustralianus* (holotype, Cui 16775) **a** basidiospores **b** basidia **c** basidioles **d** cystidioles **e** hyphae from context **f** hyphae from trama.

***Tubes*.** Generative hyphae hyaline, thin-walled, occasionally branched, 2.8–5.0 µm in diam.; skeletal hyphae dominant, yellowish brown, thick-walled with a narrow lumen, unbranched, slightly flexuous, interwoven, 2.8–3.8 µm in diam. Cystidia absent; cystidioles frequent, fusoid, hyaline, thin-walled, smooth, 7–9.7 × 3.2–3.8 µm. Basidia barrel-shaped, with four sterigmata and a basal clamp connection, 8.9–11 × 4.1–4.6 µm. basidioles of similar shape to basidia, but smaller.

***Spores*.** Basidiospores allantoid, hyaline, thin-walled, smooth, IKI–, CB–, (3.2–)3.4–4.1(–4.2) × (1.2–)1.3–1.5 µm, L = 3.62 µm, W = 1.4 µm, Q = 2.59 (n = 30/1).

#### ﻿ Nigroporus austroasianus

Taxon classificationFungiPolyporalesSteccherinaceae

Y.C. Dai, X.L. Li & Yuan Yuan sp. nov.

8469D840-058D-5572-9B0D-EA67FB15BB57

853915

Figs 5 , 6

##### Holotype.

China • Yunnan Province, Mengla County, Shangyong Nature Reserve, on rotten angiosperm wood, 20 August 2019, *Dai 20632* (BJFC 032299).

##### Etymology.

*Austroasianus* (Lat.): refers to the distribution of the species in South Asia.

##### Description.

***Basidiomata***. Annual, pileate, a few imbricate, leathery and without odor or taste when fresh, becoming corky and light in weight upon drying. Pilei semicircular to spathulate, projecting up to 4 cm, 6 cm wide and 1.4 mm thick at center. Pileal surface grayish violet to dark violet when fresh, becoming grayish brown upon drying; margin thin and sharp, usually lobed. Pore surface flesh pink to lavender when fresh, becoming fawn when bruised, pale mouse gray when dry, sterile margin distinct, cream when dry, up to 1.1 mm wide; pores round to angular, 10–13 per mm; dissepiments thin, entire to lacerate. Context vinaceous to reddish brown, corky when dry, up to 0.4 mm thick. Tubes concolorous with pore surface, corky when dry, up to 1 mm long.

**Figure 5.**
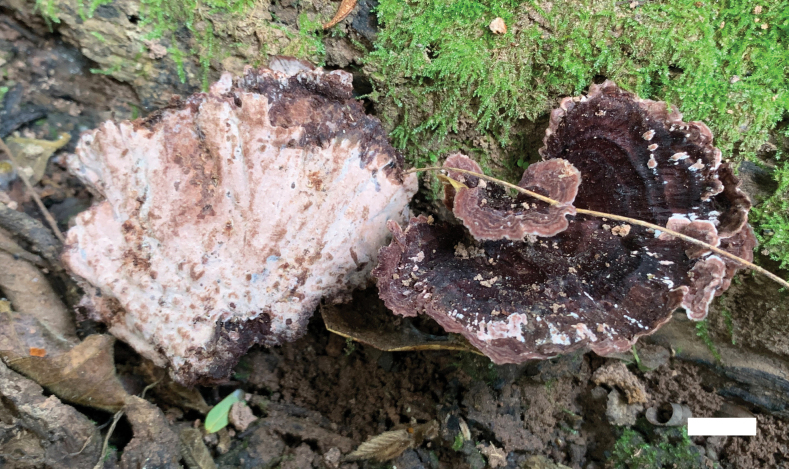
Basidiomata of *Nigroporusaustroasianus* (holotype, Dai 20632). Scale bar: 1 cm.

***Hyphal structure*.** Hyphal system dimitic; generative hyphae bearing clamp connections; skeletal hyphae IKI–, CB–; tissues darkening in KOH.

***Context*.** Generative hyphae frequent, hyaline to pale yellow, thin- to slightly thick-walled, occasionally branched, 2–4 µm in diam.; skeletal hyphae dominant, pale yellow, thick-walled with a wide lumen, unbranched, slightly flexuous, interwoven, 2.5–4 µm in diam.

**Figure 6.**
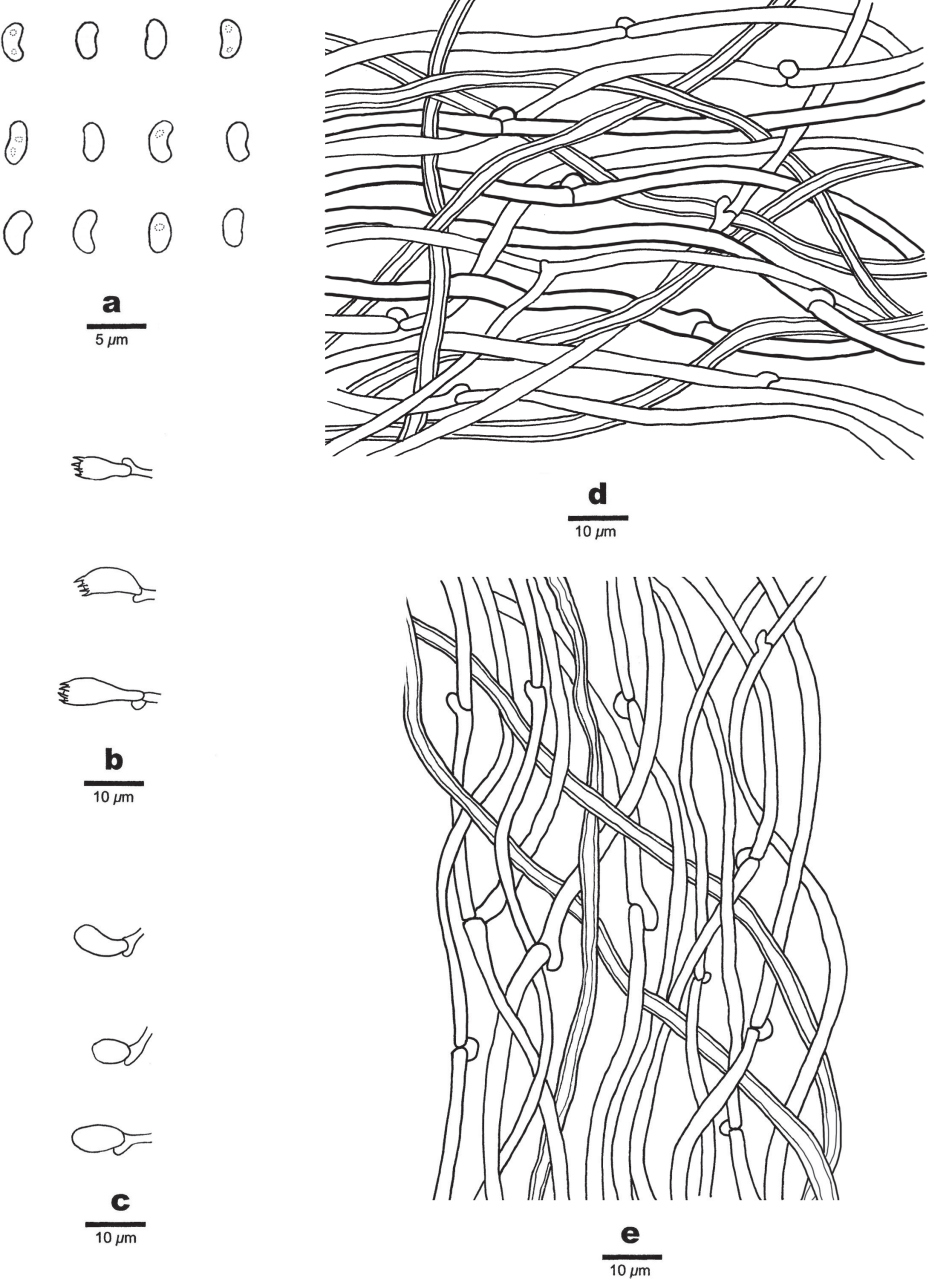
Microscopic structures of *Nigroporusaustroasianus* (holotype, Dai 20632) **a** basidiospores **b** basidia **c** basidioles **d** hyphae from context **e** hyphae from trama.

***Tubes*.** Generative hyphae dominant, hyaline, thin-walled, occasionally branched, 2.5–4.5 µm in diam.; skeletal hyphae pale yellow, slightly thick-walled with a wide lumen, unbranched, slightly flexuous, interwoven, 3.5–6.5 µm in diam. Cystidia and cystidioles absent. Basidia pyriform to barrel-shaped, with four sterigmata and a basal clamp connection, 7–12 × 3.5–4.5 µm. Basidioles barrel-shaped, smaller than basidia.

***Spores*.** Basidiospores allantoid, hyaline, thin-walled, smooth, occasionally with one or two small guttules. IKI–, CB–, (2.1–)3–4.1(–4.2) × (1.1–)1.5–2(–2.2) µm, L = 3.21 µm, W = 1.63 µm, Q = 1.83–2.01 (n = 90/3).

##### Additional specimen examined

**(*paratype*).** China • Guangxi Zhuang Autonomous Region, Leye County, Yachang Orchid Nature Reserve, stump of *Pinusyunnanensis*, 29 June 2024, *Dai 28512* (BJFC048771). Malaysia • Selangor, Kota, Damansara, Community Forest Reserve, on rotten angiosperm wood, 16 April 2018, *Dai 18594* (BJFC 026882).

#### ﻿ Nigroporus subvinosus

Taxon classificationFungiPolyporalesSteccherinaceae

Y.C. Dai, X.L. Li & Yuan Yuan sp. nov.

A10963F7-91FA-5EB3-8273-967F7B5F321B

853916

Figs 7 , 8

##### Holotype.

China • Xizang Autonomous Region, Linzhi, Motuo County, on rotten angiosperm wood, 24 October 2023, *Dai 26787* (BJFC 044337).

##### Etymology.

*Subvinosus* (Lat.): refers to the species resembling *Nigroporus vinosus*.

##### Description.

***Basidiomata*.** Annual, pileate, usually solitary, leathery and without odor or taste when fresh, becoming woody hard and light in weight upon drying; pilei semicircular to flabelliform, projecting up to 3.9 cm, 3.7 cm wide and 3 mm thick at base. Pileal surface dark grayish blue, vinaceous gray to black when fresh, become umber to fawn upon drying, concentrically zonate, glabrous, margin thin, cream. Pore surface flesh pink to brownish vinaceous when fresh, becoming fawn when bruised, reddish brown when dry; sterile margin distinct, white when dry, up to 2.3 mm wide; pores round to angular, 9–11 per mm; dissepiments thin, entire to lacerate. Context fawn to reddish brown, corky when dry, up to 1.7 mm thick. Tubes concolorous with pore surface, corky when dry, up to 1.3 mm long.

**Figure 7.**
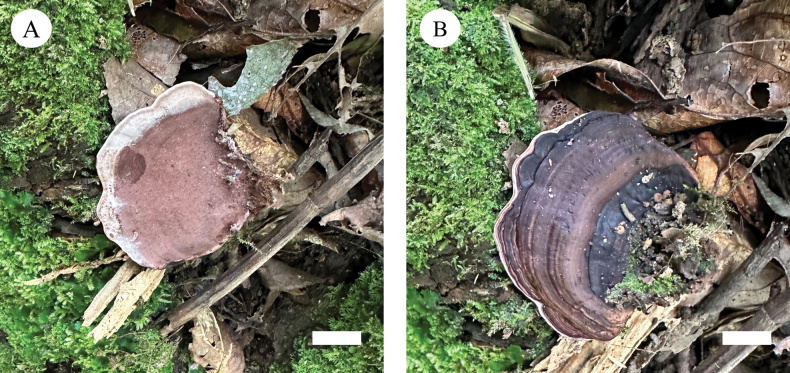
Basidiomata of *Nigroporussubvinosus* (holotype, Dai 26787). Scale bars: 1 cm (**a, b**).

***Hyphal structure*.** Hyphal system dimitic; generative hyphae bearing clamp connections; skeletal hyphae IKI–, CB–; tissues darkening in KOH.

***Context*.** Generative hyphae hyaline, thin- to slightly thick-walled, occasionally branched, 3–4.4 µm in diam.; skeletal hyphae dominant, yellowish brown, thick-walled with a narrow lumen to subsolid, unbranched, slightly flexuous, interwoven, 3.3–4.5 µm in diam.

**Figure 8.**
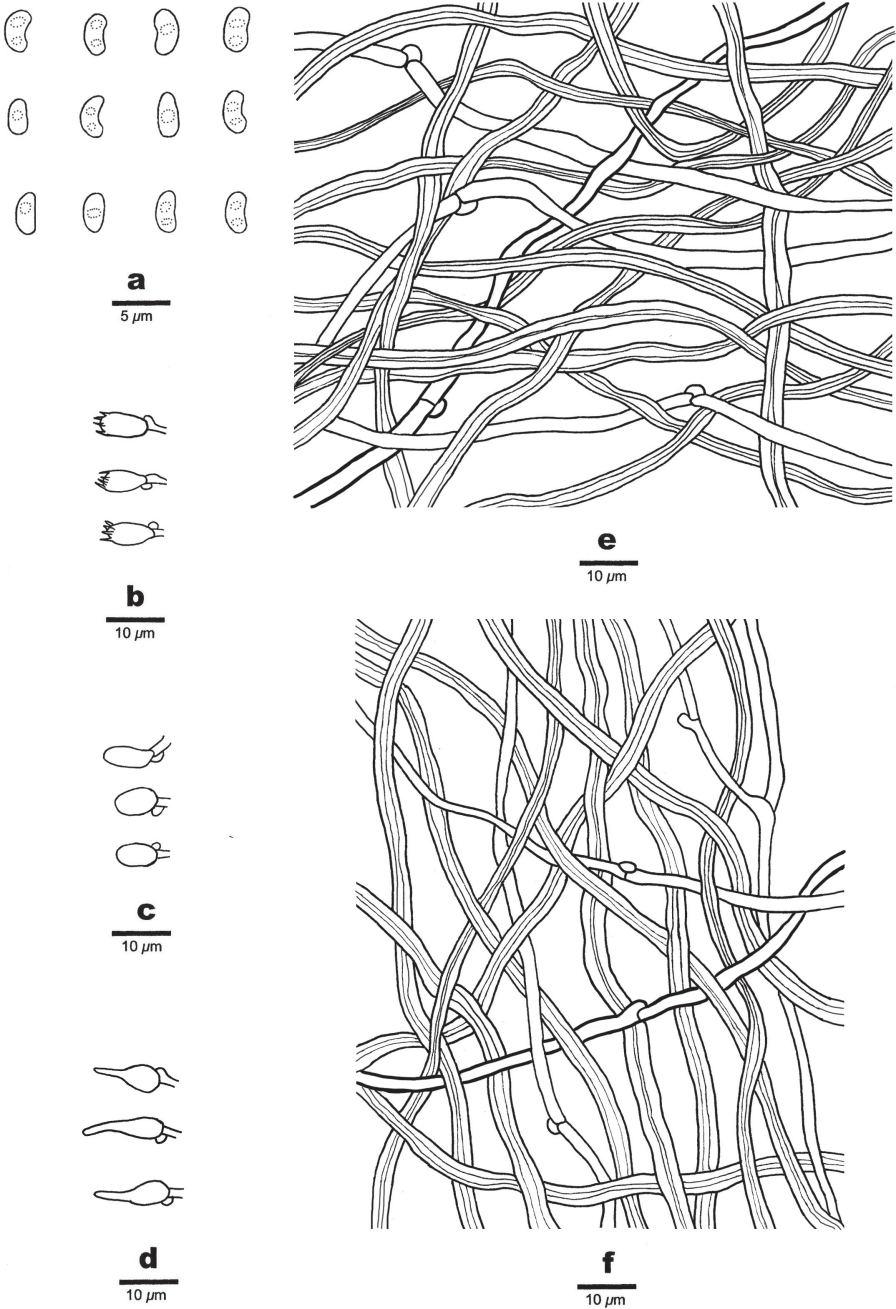
Microscopic structures of *Nigroporussubvinosus* (holotype, Dai 26787) **a** basidiospores **b** basidia **c** basidioles **d** cystidioles **e** hyphae from context **f** hyphae from trama.

***Tubes*.** Generative hyphae hyaline, thin- to slightly thick-walled, occasionally branched, 2.6–4.4 µm in diam.; skeletal hyphae dominant, yellowish brown, thick-walled with a narrow lumen to subsolid, unbranched, slightly flexuous, interwoven, 2.4–5 µm in diam. Cystidia absent; cystidioles frequent, fusoid, hyaline, thin-walled, smooth, 10–18 × 3–5 µm. Basidia barrel-shaped, with four sterigmata and a basal clamp connection, 6–10 × 3–4 µm. Basidioles of similar shape to basidia, but smaller.

***Spores*.** Basidiospores allantoid, hyaline, thin-walled, smooth, usually with one or two guttules, IKI–, CB–, (2.9–)3–4.5(–5) × (1.4–)1.5–2.1(–2.3) µm, L = 3.7 µm, W = 1.78 µm, Q = 2.04–2.09 (n = 240/8).

##### Additional specimens examined

**(*paratypes*).** China • Guangxi Zhuang Autonomous Region, Shangsi County, Natura Subsidium magnum montem National, on dead angiosperm tree, 26 May 2024, *Dai 27441* (BJFC047701). • Hainan Province, Baisha Li Autonomous County, Qingsong, on rotten wood of *Pinuslatteri*, 10 June 2023, *Dai 25154* (BJFC 042706). • Sichuan Province, Yanyuan County, on stump of *Pinusyunnanensis*, 15 August 2019, *Cui 17526* (BJFC 034385). • Yunnan Province, Baoshan City, Gaoligongshan Nature Reserve, on angiosperm stump, 7 November 2019, *Cui 18097* (BJFC 034956), *Cui 18104* (BJFC 034963). • Puer, Puer Forest Park, Xiniuping Scenic Spot, on rotten angiosperm wood, 17 August 2019, Dai *20445* (BJFC 032113). • Yingjiang County, Tongbiguan Nature Reserve, on angiosperm stump, 30 October 2012, *Dai 13136* (BJFC 013353).

#### ﻿ Nigroporus yunnanensis

Taxon classificationFungiPolyporalesSteccherinaceae

Y.C. Dai, X.L. Li & Yuan Yuan sp. nov.

AE07B89F-B79E-5F22-853A-05CC44B3EEF4

853917

Figs 9 , 10

##### Holotype.

China • Yunnan Province: Baoshan County, Baihualing Forest Park, on fallen angiosperm trunk, 21 September 2018, *Dai 19116* (BJFC 027585).

##### Etymology.

*Yunnanensis* (Lat.): refers to the species being found in Yunnan Province, southwest China.

##### Description.

***Basidiomata*.** Annual, effused-reflexed to pileate, leathery and without odor or taste when fresh, becoming woody hard upon drying, up to 8.5 cm long, 4 cm wide when resupinate; pilei semicircular, projecting up to 1 cm, 1.5 cm wide and 2.5 mm thick at base. Pileal surface brown when fresh, becoming liver brown to dark brown upon drying, concentrically zonate, glabrous, margin thin. Pore surface vinaceous to brownish vinaceous when fresh, become purplish date when bruised, russet when dry; sterile margin distinct, white when fresh, and cream when dry, up to 0.5 mm wide; pores round to angular, 7–10 per mm; dissepiments thin, lacerate. Context liver brown, hard corky when dry, up to 1.5 mm thick. Tubes concolorous with pore surface, corky when dry, up to 1 mm long.

**Figure 9.**
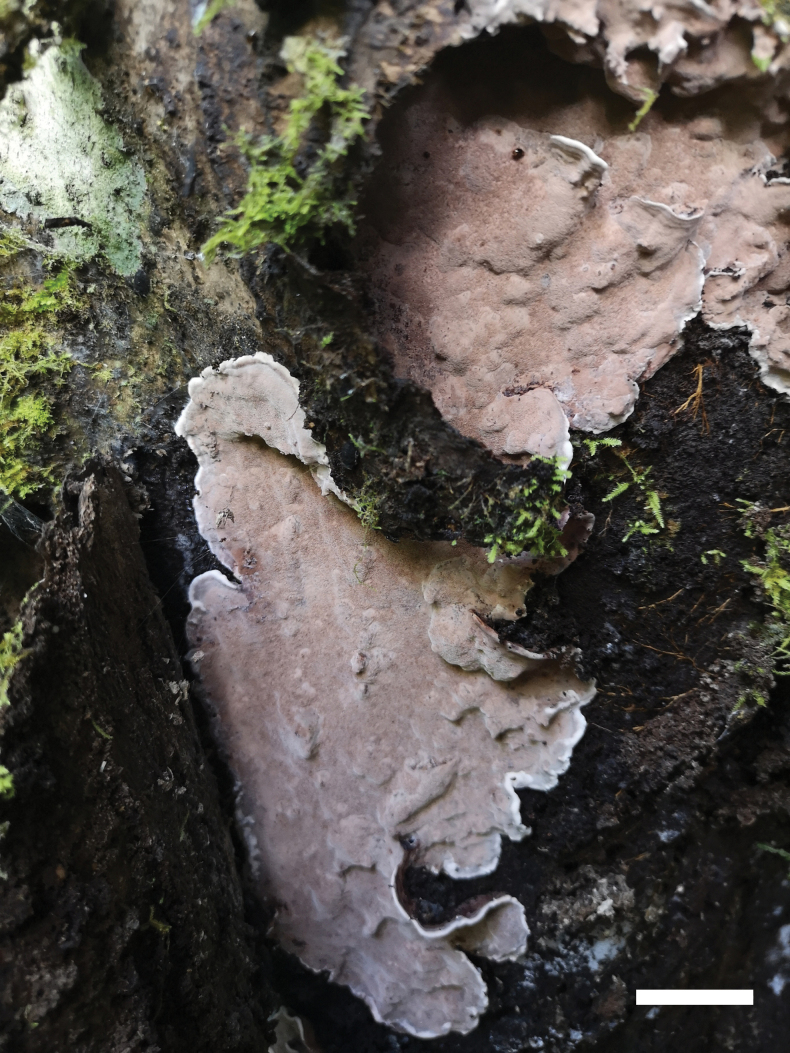
Basidiomata of *Nigroporusyunnanensis* (holotype, Dai 19116). Scale bar: 1 cm.

***Hyphal structure*.** Hyphal system dimitic; generative hyphae bearing clamp connections; skeletal hyphae IKI–, CB–; tissues darkening in KOH.

***Context*.** Generative hyphae hyaline, thin-walled, occasionally branched, 1.5–4 µm in diam.; skeletal hyphae dominant, yellowish brown, slightly thick-walled with a wide lumen, unbranched, slightly flexuous, interwoven, 3–4.5 µm in diam.

**Figure 10.**
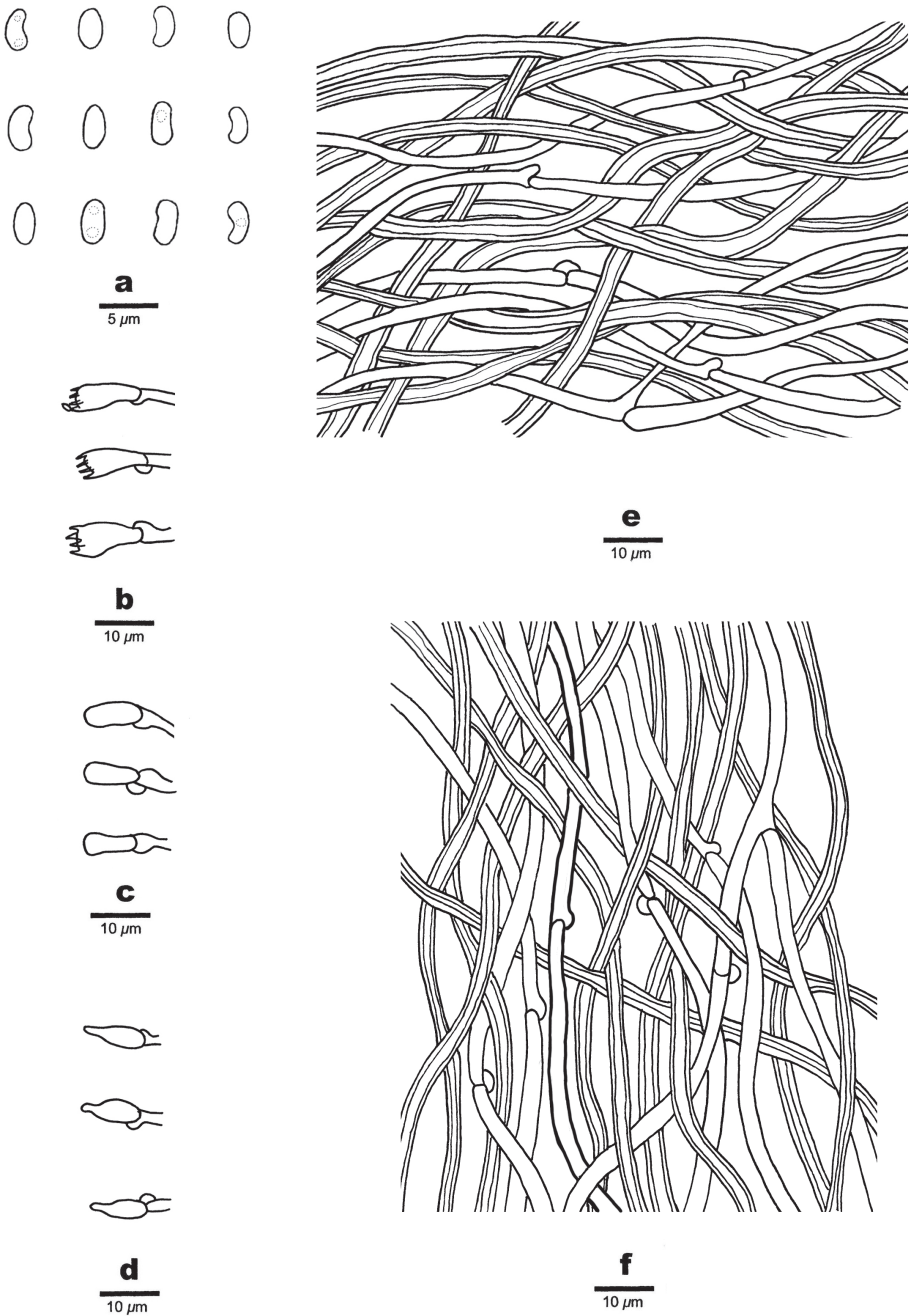
Microscopic structures of *Nigroporusyunnanensis* (holotype, Dai 19116) **a** basidiospores **b** basidia **c** basidioles **d** cystidioles **e** hyphae from context **f** hyphae from trama.

***Tubes*.** Generative hyphae hyaline, thin- to slightly thick-walled, occasionally branched, 2.5–4.5 µm in diam.; skeletal hyphae dominant, yellowish brown, thick-walled with a wide to narrow lumen, unbranched, slightly flexuous, interwoven, 4–5 µm in diam. Cystidia absent; cystidioles present, fusoid, hyaline, thin-walled, smooth, 7.5–11 × 2.5–4 µm. Basidia barrel-shaped, with four sterigmata and a basal clamp connection, 5–7.5 × 3–4.5 µm; basidioles of similar shape to basidia, but smaller.

***Spores*.** Basidiospores allantoid, hyaline, thin-walled, smooth, occasionally with one or two guttules. IKI–, CB–, (3.8–)4–4.5(–5) × (1.8–)1.9–2.2(–2.5) µm, L = 4.19 µm, W = 2.03 µm, Q = 2.06–2.09 (n = 90/3).

##### Additional specimens examined

**(*paratypes*).** China • Yunnan Province, Pingbian County, Daweishan National Forest Park, on fallen angiosperm branch, 27 June 2019, *Dai 19870* (BJFC 031544). • Tengchong County, Gaoligongshan, Dahaoping, on dead angiosperm tree, 10 November 2019, *Cui 18205* (BJFC 035064).

## ﻿Discussion

In the present study, phylogenetic analyses on the Steccherinaceae using a 2-gene sequence dataset (Fig. 1) and on *Nigroporus* using a 3-gene sequence dataset (Fig. 2) were carried out. *Nigroporus* is monophyletic and closely related to *Trullella* Zmitr. However, *Trullella* has more or less cream-colored basidiomata, a monomitic hyphal structure in context, hyaline and cyanophilous skeletal hyphae, and leptocystidia (Zmitrovich 2018), while *Nigroporus* has pinkish to bluish gray basidiomata, a dimitic hyphal system in context, fuliginous and acyanophilous skeletal hyphae, and the absence of leptocystidia.

Phylogenetically, *Nigroporusaustralianus*, *N.austroasianus*, *N.subvinosus* and *N.yunnanensis* are closely related to *N.vinosus* (Figs 1, 2), and have more or less similar macromorphology, that is why the four new species were treated as the *N.vinosus*. However, the nucleotide differences in the ITS regions among these species are more than 2.5%. Morphologically, *N.australianus* is different from *N.austroasianus*, *N.subvinosus*, *N.vinosus* and *N.yunnanensis* by its indistinct sterile margin and narrower basidiospores (1.3–1.5 µm, Q = 2.59 vs. 1.5–2.2 µm, Q = 1.83–2.09). *N.austroasianus* differs from *N.subvinosus*, *N.vinosus* and *N.yunnanensis* by smaller pores (10–13 per mm vs. 7–11 per mm), generative hyphae dominant in tube trama, and lacking cystidioles, while skeletal hyphae are dominant in tube trama and cystidioles are present in the latter three species. *Nigroporussubvinosus* is distinguished from *N.vinosus* by basidiospores with guttules and subsolid skeletal hyphae. *Nigroporussubvinosus* differs from *N.yunnanensis* by thicker basidiomata (> 3 mm vs. < 2.5 mm). *Nigroporusyunnanensis* is different from *N.vinosus* by shorter cystidioles (7.5–11 µm vs. 12–18 µm). Among the existing species of *Nigroporus* without DNA available, the four new species are readily distinguished from *N.stipitatus* by having pileate basidiomata (Douanla-Meli et al. 2007), and they are different from *N.macroporus* and *N.ussuriensis* by having smaller pores (7–13 per mm vs. 1–2 per mm in *N.macroporus* vs. 5–7 per mm in *N.ussuriensis*, Ryvarden and Iturriaga 2003). Furthermore, basidiospores of our new species are allantoid without tapering apex, while the basidiospores of *N.ussuriensis* are cylindric with a distinctly tapering apex (Dai and Niemelä 1995). Despite the phylogenetic results showing a distinct genetic distance between the Malaysian material (Dai 18594) and the Chinese materials (Dai 20632 and Dai 28512, Figs 1, 2), the three specimens have very similar morphology, and they represent a single species, *N.austroasianus*.

*Polyporustristis* Lév. was described from Indonesia by Léveillé (1846), and it was considered a synonym of *Nigroporusvinosus*. Our new species *N.austroasianus* is derived from *N.vinosus*, and was found in Malaysia and tropical to subtropical China (South Asia). Both names may represent a single species, but *Polyporustristis* is nomenclature illegitimate because *Polyporustristis* Pers. 1825 (=*Trametestristis* (Pers.) Roum.) had priority. So, we describe the tropical Asia taxon as *N.austroasianus*.

### ﻿Key to accepted species of *Nigroporus*

**Table:** 

1	Basidiomata stipitate	***N.stipitatus***
–	Basidiomata pileate to resupinate	**2**
2	Pores 1–2 per mm; South American species	***N.macroporus***
–	Pores > 3 per mm; pantropical species	**3**
3	Cystidioles absent	**4**
–	Cystidioles presence	**5**
4	Pores 5–7 per mm	***N.ussuriensis***
–	Pores 10–13 per mm	***N.austroasianus***
5	Pilei < 2.5 mm thick at base	***N.yunnanensis***
–	Pilei > 2.5 mm thick at base	**6**
6	Basidiospores < 1.5 µm wide	***N.australianus***
–	Basidiospores > 1.5 µm wide	**7**
7	Basidiospores with one or two guttules and skeletal hyphae subsolid	***N.subvinosus***
–	Basidiospores without guttule and skeletal hyphae with a wide lumen	***N.vinosus***

## Supplementary Material

XML Treatment for Nigroporus australianus

XML Treatment for Nigroporus austroasianus

XML Treatment for Nigroporus subvinosus

XML Treatment for Nigroporus yunnanensis

## ﻿Additional information

### Author ORCIDs

Xiang-Lin Li https://orcid.org/0009-0007-4364-6994

Yu-Cheng Dai https://orcid.org/0000-0002-6523-0320

Zhan-Bo Liu https://orcid.org/0000-0002-3894-5398

Hong-Gao Liu https://orcid.org/0000-0002-9508-3245

Yuan Yuan https://orcid.org/0000-0001-6674-9848

### Data availability

All of the data that support the findings of this study are available in the main text.
